# Biomonitoring of Trace Elements in Hair of Schoolchildren Living Near a Hazardous Waste Incinerator—A 20 Years Follow-Up

**DOI:** 10.3390/toxics7040052

**Published:** 2019-10-01

**Authors:** Roser Esplugas, Montse Mari, Montse Marquès, Marta Schuhmacher, José L. Domingo, Martí Nadal

**Affiliations:** 1 Laboratory of Toxicology and Environmental Health, School of Medicine, IISPV, Universitat Rovira i Virgili, 43201 Reus, Catalonia, Spain; roser.esplugas@urv.cat (R.E.); montserrat.marques@urv.cat (M.M.); joseluis.domingo@urv.cat (J.L.D.); 2 Environmental Engineering Laboratory, Departament d’Enginyeria Quimica, Universitat Rovira i Virgili, 43007 Tarragona, Catalonia, Spain; montserrat.mari@urv.cat (M.M.); marta.schuhmacher@urv.cat (M.S.)

**Keywords:** Trace elements, hair, children, hazardous waste incinerator, Constantí (Catalonia, Spain)

## Abstract

Since 1998, a monitoring program is periodically performed to assess the environmental and human health impact of air chemicals potentially emitted by a hazardous waste incinerator (HWI) located in Constantí (Catalonia, Spain). In 2017, samples of hair were collected from 94 schoolchildren (aged 10–13 years) living nearby and the levels of 11 trace elements (As, Be, Cd, Cr, Hg, Mn, Ni, Pb, Sn, Tl and V) were determined. The concentrations showed the following descending order: Pb > Hg > Ni > Sn > Mn > Cr. In turn, As, Be and Tl were not detected, while Cd and V were found only in a few samples. Some metal levels were significantly, positively correlated. Some significant differences were also noticed according to the gender and the specific zone of residence. Finally, the levels of trace elements showed fluctuations through time. Cr and Pb showed a significant decrease in comparison to the concentrations obtained in the baseline study (1998). According to the current results, metal emissions from the HWI are not relevant in terms of human health impact since their levels were similar and even lower than those reported in other contaminated areas.

## 1. Introduction

To date, there is only a hazardous waste incinerator (HWI) in Spain, which is located in Constantí (Tarragona County, Catalonia). Being built in 1996–1998, it started to operate in 1999. At the building time, a large pre-operational monitoring program was initiated, not only as an additional measure of environmental control but also responding to the demands of residents and public authorities. The study was focused on two chemicals of special concern: polychlorinated dibenzo-*p*-dioxins and dibenzofurans (PCDD/Fs) as well as metals and metalloids. One of the main goals of this program was aimed at assuring that the facility would not be a relevant source of environmental pollution and its operations should not affect the health of the population living nearby. The initial surveillance program was designed to evaluate the impact on the environment, through the monitoring of soil and vegetation [1,2], while the impact on the residents was assessed through the monitoring of biological tissues such as blood, breast milk and hair [3,4,5,6,7,8]. Furthermore, the dietary intake of PCDD/Fs and metals by the local population was also evaluated [9,10].

The location of this HWI, which is situated near a chemical/petrochemical industrial complex, a municipal waste incinerator and in a zone with heavy traffic, can mean additional toxic emissions. Considering that there exist many emission sources in the area, the environmental surveillance of metals and metalloids is clearly a need for public health. The non-occupationally population is exposed to trace elements mainly through the diet including water [11], being inhalation and transdermal absorption minor exposure pathways [12]. The effects of a chronic/acute exposure to trace elements are varied, including cancer (e.g., arsenic [As], cadmium [Cd], chromium [Cr], nickel [Ni]), skin lesions (e.g., As, beryllium [Be], tin [Sn]), neurological disorders (e.g., mercury [Hg], manganese [Mn], thallium [Tl]), learning disability (e.g., lead [Pb]) or respiratory problems (e.g., vanadium [V]) among others [13,14,15,16,17]. Furthermore, it should be taking to account that the synergistic effect of the co-exposure to different metals and metalloids can also lead to cumulative adverse health effects [18,19].

The monitoring program has been continuously conducted since 1999. While information of the environmental levels of pollutants has been quite recurrent in the last 20 years [20], data on the concentrations of trace elements in the same biomonitors (i.e., hair, blood and autopsy tissues) have been updated every 4–5 years [21,22,23,24].

Despite the traditional approach for human biomonitoring is based on the analysis of blood and urine, human hair is also a very useful and valuable biological matrix [25]. The levels of metals in hair are up to 10-fold higher than those usually found in blood or urine [26]. The concentrations of heavy metals in hair can be modulated by endogenous factors including metabolic pathways, as well as exogenous impregnations such as air pollutants [27]. More interestingly, hair samples allow an easy sampling and storage, being a non-invasive method.

The biomonitoring of children is more complex than that of adults. However, results are undoubtedly of great interest [28], as children are more susceptible to metal exposure, since they have higher absorption rates in relation to their body weight. Moreover, they have low capacity of detoxification and excretion, as well as behavioural patterns that the environmental pollution can potentially affect more easily [29,30].

Being part of a large biological surveillance program, this study was aimed at identifying whether there are any health risks for the population living close to the HWI. More specifically, the purpose of the present study was to measure the concentration of As, Be, Cd, Cr, Hg, Mn, Ni, Pb, Sn, Tl and V in hair of schoolchildren who live near the facility. A detailed analysis of the correlation between metals and the differences according to the sex and specific zones of residence was also carried out. Furthermore, temporal trends were determined by comparing these data with those of previous surveys.

## 2. Materials and Methods

### 2.1. Sample Collection

**Figure 1:**
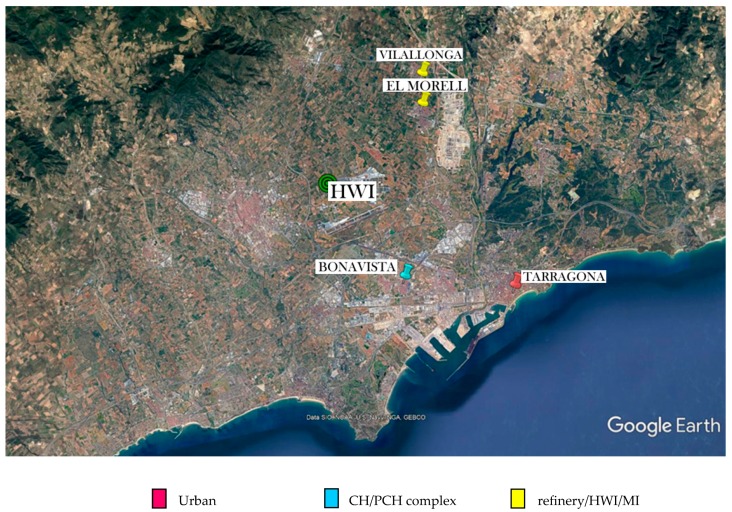
Sampling area.

Between June and September of 2017, hair samples were obtained from 94 schoolchildren (44 boys and 50 girls aged 10–13 years). About 2–3 cm of hair was cut from an area close to the occipital region of the scalp. Only naturally coloured hair was selected in order to avoid biased results. The participants were classified in 3 groups according to the area of residence: (1) urban (named as *downtown*), (2) close to an important chemical/petrochemical complex (named as *CH/PCH complex*) and (3) near a large oil refinery, the HWI and a municipal incinerator (named as *refinery/HWI/MI*) (Figure 1). A consent form according to the declaration of Helsinki was signed by the tutors of each participant. The study protocol, 07/2017, was reviewed and approved by the Ethical Committee for Human Studies of the Pere Virgili Health Research Institute (IISPV), Reus/Tarragona, Spain in March 30, 2017 [22,23].

### 2.2. Pre-treatment and Chemical Analysis

Hair samples were washed with 1% Triton X-100 (E. Merck, Darmstadt, Germany) in an ultrasounds bath for 20 minutes in order to remove external contamination. Then, the soap was removed with distilled water and samples were 3 times-washed with Milli-Q water. Around 150 mg of sample were placed in hermetic Teflon bombs and digested with 2 mL of 65% nitric acid (Suprapur, E. Merck, Darmstadt, Germany) for 12 hours at 110 °C. Samples were then filtered, diluted to 10 mL of Milli-Q water and stored at −20 °C.

The concentrations of As, Be, Cd, Cr, Hg, Mn, Ni, Pb, Sn, Tl and V in hair samples were analysed according to previous studies conducted in our laboratory [6,31,32,33] using inductively coupled plasma-mass spectrometry (ICP-MS, Perkin Elmer Elan 6000). Each sample was tested by duplicate, being *Lobster Hepatopancreas* employed as quality control (*NRC Canada*, *TORT-2*) every 5 samples. Blanks used during the digestion were also run every 5 samples. The limits of detection (LODs) were 0.03 µg/g for Be, Cd, Pb and Tl; 0.07 µg/g for As, Cr, Mn, Ni, Sn and V; and 0.13 µg/g for Hg.

### 2.3. Statistical Analysis

All data were analysed by using the statistical package SPSS 25.0. Values with a *Z*-score above 2.5 and under −2.5 were considered as outliers. In addition, a visual inspection of the boxplot was also conducted to verify these outliers. Non-detected levels of metals were assumed as to be one-half of the respective LOD (ND = ½ LOD). Only trace elements with values 70% (or more) above the LOD were considered for further statistical evaluation. To assess the distribution of the values, the Kolmogorov-Smirnov test was used. Correlations between metal concentrations were performed employing the Spearman correlation coefficient. The Student’s t-test was used to compare differences of metal levels between boys and girls. In turn, ANOVA and subsequent T3 Dunnett’s post-hoc tests were employed to assess differences between groups according to the zones of residence and the significance in the temporal evolution. The level of statistical significance was established at *p* < 0.05.

## 3. Results

The concentrations of the 11 analysed elements in 94 samples of hair from schoolchildren living in Tarragona County are shown in Table 1. Arsenic, Be and Tl were not detected, whereas Cd and V were only detected in 37 and 23 samples, respectively. In previous campaigns of the monitoring program, traces of Cd and V were only found in very few samples of hair [6,31,32,33]. Although the information relative to these elements is shown, they are excluded from the statistical analysis.

Lead presented the highest mean concentration (1.44 µg/g), ranging from undetected values to 11.9 µg/g. Mercury also presented relatively high levels, with a mean and a range of 0.73 and 0.13–2.70 µg/g, respectively. The mean concentrations of Sn, Mn and Cr were 0.41, 0.30 and 0.14 µg/g, respectively. Finally, Cd and V were detected only in a few samples. Both metals exhibited the lowest concentrations (0.04 and 0.07 µg/g, respectively). When evaluating the correlation between metal levels (Table 2), a weak but significant positive correlation, was found between Mn and Ni, Pb (*p* < 0.01) and Sn (*p* < 0.05) and also between Ni and both Cr and Sn (*p* < 0.01).

**Figure 2:**
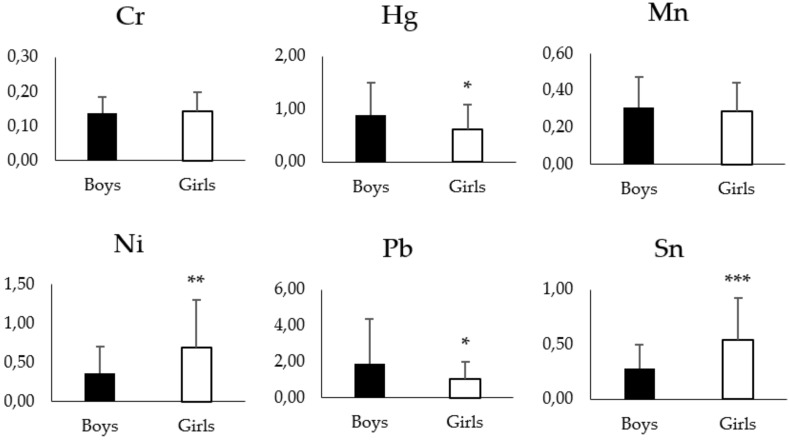
Metal concentrations (µg/g) in hair of schoolboys (*n* = 44) and girls (*n* = 50) living in Tarragona County (2017). Data are given as mean ± SD. Statistics: non-parametric t-test. Significant differences between both groups at: *p < 0.05, **p < 0.01 and ***p < 0.001.

When metal concentrations were assessed according to the sex of the schoolchildren, significant differences were found in the levels of Hg, Ni, Pb and Sn (Figure 2). Thus, girls exhibited significant lower levels of Hg (0.61 µg/g vs. 0.88 µg/g, (*p* < 0.05)) and Pb (1.03 µg/g vs. 1.89 µg/g, *p* < 0.05) and significant higher concentrations of Ni (0.69 µg/g vs. 0.36 µg/g, *p* < 0.01) and Sn (0.54 µg/g vs. 0.28 µg/g, *p* < 0.001) than boys. Finally, Cd, Cr and Mn did not show significant differences between girls and boys.

Mercury was identified as the only element showing significant differences in the levels in hair according to the specific zones of residence (Table 3). Significantly higher concentrations of Hg were found in the children living in Tarragona downtown with respect to those living in the remaining two evaluated areas (*p* < 0.01), both of them with industrial characteristics.

The temporal trends of metal concentrations in hair of schoolchildren are summarized in Table 4. The results of the current study are compared with those of previous surveys, including the baseline study (1998) and the intermediate campaigns (2002, 2007 and 2012). A significant decrease of Cd in recent years was noted when compared to the baseline results (*p* < 0.001). Chromium levels significantly increased in 2007 and 2012 in comparison to previous years (*p* < 0.001) but the concentration of this element notably decreased in 2017 (*p* < 0.001), being this reduction significant in contrast to the previous surveys. Despite Ni concentrations were significantly lower in 2002 (*p* < 0.001), they have remained nearly constant through time. Furthermore, although Pb concentrations decreased significantly after the baseline survey (*p* < 0.001), in 2017 they raised again, being significantly higher than those observed in 2007 and 2012 (*p* < 0.01). Tin showed significantly lower concentrations in the period 2002–2012 (*p* < 0.001), while current values are comparable to those found in 1998. Mercury was the only element which did not exhibit significant differences between surveys. Finally, V was found at relatively low concentrations, being detected in only 23 samples in 2017 but contrasting with data from previous surveys, where it was never detected.

## 4. Discussion

Some metals were significantly and positively correlated: Mn with Ni, Pb and Sn and also Ni with Cr and Sn. This kind of correlations has been previously reported in the scientific literature. In Russia, Semenova et al. [34] also found a positive correlation between Pb and Mn in hair of children living in the vicinity of abandoned mines in the South Urals, while Drobyshev et al. [35] reported positive correlations of Al, Cu, Fe, Ni and V, when analysing the concentrations of trace elements in children (aged 7–9) of St. Petersburg.

The body growth, physiology, the presence of specific sexual hormones and metabolizing enzymes, along with lifestyle and physical activity contribute to exhibit differences in the accumulation and excretion processes of metals between boys and girls [36]. Sex can play a role, being suggested that females are more vulnerable to exposure to trace metals than males, particularly at higher levels of exposure [37]. Some authors have even reported higher concentrations of trace elements in hair of girls than in boys [14,38]. In agreement with these findings, girls living in Tarragona County showed significantly higher levels of Ni and Sn than boys. However, Hg and Pb were found at lower levels in girls, when compared to boys. Further research is required to better understanding the sex differences in metal levels in hair, especially highlighting if changes can be applied to all the trace elements or there exist variabilities among metals.

It has been largely described that the profile of hair metal composition depends on local environmental conditions [39]. When we analysed the differences in metal levels between zones of residence, it was observed that children living in Tarragona downtown presented higher levels of Hg than those living near the CH/PCH complex or near the refinery/HWI/MI. Since the environmental concentrations of Hg are not increased in this area according to data from soils and vegetation [40,41], traffic could be even more important than the industrial activity, in terms of Hg exposure. However, it must be remarked that dietary intake plays a key role in human exposure to metals. Food consumption is the most contributive pathway of exposure to metals and metalloids [42]. As suggested by Castaño et al. [15], the relationship between the zone of residence and Hg concentrations could be attributed to the nutritional habits of children, which are invariably different according to their socio-economic status.

The temporal profile of metal concentrations in hair of schoolchildren has revealed some interesting findings. Thus, the significant reduction of Pb since 1998 may be attributed to their removal from gasoline, as this element -as an additive- was banned in 2001. In fact, the benefits of this legislative measure, in terms of environmental pollution by Pb, have been largely observed [43]. However, an accurate assessment of the temporal trends of metal concentrations in hair should be conducted also using data from other biological tissues, which are not currently available. Furthermore, changes in the dietary habits of the population living in Tarragona County should be also considered. In 2012, an increase in the dietary exposure to Hg and Cr was reported [44]. In turn, a spectacular decrease in the intake of both elements occurred recently (unpublished data), which was also found for PCDD/Fs [45].

The concentrations of trace elements in hair of schoolchildren were compared with those of recent studies found in the scientific literature (Table 5). Most of these investigations are focused on areas with important industrial or mining activities or even in urban zones [11,14,26,30,34,35,36,37,46,47]. The different results might be attributed to the differences among the respective geographical areas. Furthermore, despite the mean Cr concentration is similar to most levels found in recent literature, Cr concentrations in the urban area of Madrid were approximately 5-fold higher than those of the present study [46]. The mean level of Hg was also higher than the concentrations found in other locations, such as toxic waste disposal sites of Russia [35] and different urban areas [14,47]. In contrast, Hg concentrations in children from Tarragona County were lower than those found in other Spanish studies focused on assessing urban and industrial/mining areas [26,46]. The current Mn values were lower than elsewhere, differing notably from those found in Sardinia (Italy) and Russia [34,35,37]. In contrast, higher Ni levels were found when our data are compared with those of other studies, excepting those reported by Xie et al. [30] in Shaoguan Guangdong (China) and Evrenoglou et al. [14] in Athens (Greece). In turn, Pb levels were higher than those found in hair of children living in some mining, volcanic, sub-urban and urban areas [14,26,34,36,47] but lower than values from children living near certain mining zones, as well as near to toxic waste disposal sites and cement plants [11,30,34,35,37]. The individual characteristics of the geographic areas where the children live would probably be the responsible of the observed variability in Ni and Pb levels among studies.

Thus, the trace element levels obtained in our study, which are similar and even lower than those reported in other contaminated areas, suggest that metal emissions from the HWI are not relevant in terms of human health impact. Nevertheless, additional studies in combination with information from other biological tissues, as well as the dietary intake of metals, are clearly needed for a complete identification of potential health risks.

## 5. Conclusions

The presence of Cd, Pb, Hg, Ni, Sn, Mn, Cr and V in hair of schoolchildren living near the HWI of Constantí (Tarragona County, Catalonia, Spain) was confirmed in this study. Some significant differences according to the sex were noted, while Hg was the only metal with significant differences according to the zone of residence of the schoolchildren. The levels of Cr and Pb decreased since 1998, when the biomonitoring program was started and despite Mn, Ni, Pb and Sn showed higher levels in 2017 when compared to some previous surveys, their concentrations were even lower than those reported in recent literature. The present results indicate that the current emissions of metals by the HWI do not pose direct health risks of immediate concern. The follow-up monitoring program should be continued in order to assure there are no changes in human exposure to trace elements in the vicinity of the HWI.

## Figures and Tables

**Table 1:** Metal concentrations (µg/g) in hair of 94 school children living in Tarragona County (2017).

Metal	Mean ± SD	Median	Recovery (%)	Interquartile Range	LOD	Maximum
As	ND	-	81.6	-	0.07	
Be	ND	-	42.3	-	0.03	
Cd	0.04 ± 0.05	0.02	85.0	0.02	0.03	0.44
Cr	0.14 ± 0.05	0.13	92.3	0.00	0.07	0.44
Hg	0.73 ± 0.56	0.59	86.9	0.62	0.13	2.70
Mn	0.30 ± 0.16	0.25	86.4	0.23	0.07	0.77
Ni	0.54 ± 0.53	0.36	73.3	0.48	0.07	2.66
Pb	1.44 ± 1.89	0.93	73.9	1.52	0.03	11.86
Sn	0.41 ± 0.34	0.32	47.1	0.42	0.07	1.64
Tl	ND	ND	88.9	-	0.03	-
V	0.07 ± 0.08	0.07	104.2	0.16	0.07	0.43

ND = not detected, LOD = limit of detection. Data are given as mean ± SD.

**Table 2:** Correlations between metal concentrations in hair of 94 schoolchildren living in Tarragona County (2017).

Metal	Cr	Hg	Mn	Ni	Pb	Sn	V
Cd	0.085	0.028	0.261	0.345	0.533	0.375	0.181
Cr		0.061	0.177	0.271 **	0.198	0.091	0.180
Hg			−0.057	−0.130	0.077	0.047	0.164
Mn				0.405 **	0.291 **	0.234 *	0.128
Ni					0.158	0.299 **	0.286
Pb						0.158	0.106
Sn							0.268

Spearman correlation coefficient (r) is shown. * and ** means significant differences at *p* < 0.05 and *p* < 0.01 (bilateral), respectively.

**Table 3:** Metal concentrations (µg/g) in hair of school children living in Tarragona County according to the specific zones of residence (2017).

Metal	Downtown	CH/PCH Complex	Refinery/HWI/MI
As	ND	ND	ND
Be	ND	ND	ND
Cd	0.03 ± 0.03	0.04 ± 0.09	0.04 ± 0.03
Cr	0.14 ± 0.05	0.15 ± 0.07	0.13 ± 0.03
Hg	1.07 ± 0.64 ^a^	0.54 ± 0.45 ^b^	0.56 ± 0.40 ^b^
Mn	0.26 ± 0.15	0.29 ± 0.20	0.33 ± 0.15
Ni	0.42 ± 0.41	0.55 ± 0.61	0.63 ± 0.56
Pb	1.08 ± 0.85	1.55 ± 2.93	1.73 ± 1.82
Sn	0.47 ± 0.37	0.27 ± 0.26	0.44 ± 0.34
Tl	ND	ND	ND
V	0.10 ± 0.10	0.07 ± 0.08	0.04 ± 0.03

ND = not detected. Data are given as mean ± SD. Statistics: ANOVA and T3 Dunnett’s post-hoc test. Data not showing a common superscript (^a, b, c^) indicate significant differences between zones (downtown, CH/PCH complex and refinery/HWI/MI) at *p* < 0.05.

**Table 4:** Metal concentrations (µg/g) in hair of school children living in Tarragona County obtained in the studies conducted in 1998, 2002, 2007, 2012 and 2017.

Metal	1998	2002	2007	2012	2017
**As**	ND	ND	ND	ND	ND
**Be**	ND	ND	ND	ND	ND
**Cd**	0.18 ± 0.14 ^a^	ND	0.02 ± 0.01 ^b^	0.02 ± 0.01 ^b^	0.04 ± 0.05
**Cr**	0.36 ± 0.52 ^a^	0.37 ± 0.21 ^a^	1.31 ± 1.14 ^b^	0.98 ± 0.22 ^b^	0.14 ± 0.05 ^c^
**Hg**	0.67 ± 0.42	0.70 ± 0.45	0.56 ± 0.53	0.58 ± 0.23	0.73 ± 0.56
**Mn**	0.26 ± 0.17 ^ac^	0.16 ± 0.23 ^b^	0.21 ± 0.24 ^ab^	0.20 ± 0.23 ^b^	0.30 ± 0.16 ^c^
**Ni**	0.65 ± 0.54 ^a^	0.27 ± 0.22 ^b^	0.48 ± 0.58 ^a^	0.53 ± 0.73 ^a^	0.54 ± 0.53 ^a^
**Pb**	5.81 ± 3.86 ^a^	0.86 ± 2.02 ^bc^	0.58 ± 0.68 ^b^	0.63 ± 0.78 ^b^	1.44 ± 1.89 ^c^
**Sn**	0.37 ± 0.45 ^a^	0.13 ± 0.10 ^b^	0.16 ± 0.18 ^b^	0.20 ± 0.29 ^b^	0.41 ± 0.34 ^a^
**Tl**	ND	ND	ND	ND	ND
**V**	ND	ND	ND	ND	0.07 ± 0.08

ND= not detected. Data are given as mean ± SD. Statistics: ANOVA and T3 Dunnett’s post-hoc test. Data not showing a common superscript (^a, b, c^) indicate significant differences between years (1998, 2002, 2007, 2012 and 2017) at *p* < 0.05.

**Table 5:** Summary of recently published metal concentrations (µg/g) in hair of children in different countries.

City, Country	Zone	Age (years)	Sex	As	Be	Cd	Cr	Hg	Mn	Ni	Pb	Sn	Tl	V	Reference
Constantí, Tarragona, Spain	HWI, industrial, urban	7–13	Both	ND	ND	0.04	0.14	0.73	0.30	0.54	1.44	0.41	ND	0.07	Present study
Karabash, Russia *	copper smelter	14	Both	0.06		0.07	0.16		0.72	0.21	1.96				Skalny et al. [11]
Varna, Russia *	control locations	14	Both	0.06		0.04	0.26		0.53	0.23	1.43				Skalny et al. [11]
Tomino, Russia *	control locations	14	Both	2.04		0.12	0.14		0.60	0.30	5.44				Skalny et al. [11]
Kifisia Athens, Greece	urban	11–12	Both	0.031		0.03		0.49		0.92	1.36				Evrenoglou et al. [14]
Philadelphia, Athens, Greece	urban	11–12	Both	0.035		0.03		0.52		1.38	3.31				Evrenoglou et al. [14]
Kryoneri, Athens, Greece	sub-urban	11–12	Both	0.026		0.03		0.36		0.62	0.80				Evrenoglou et al. [14]
Huelva, Spain	living near industrial/mining areas	6–9	Both	0.07		<0.003		1.28	0.26		<0.09				Molina-Villalba et al. [26]
Shaoguan Guangdong, Tielong, China	cement plant and ex-mining area	≤15	Both	1.13		0.19	0.17			1.21	10.88				Xie et al. [30]
Rural settlements Tubinsk, Russia	vicinity of abandoned mines in South Urals	7–14	Both			0.05			1.43	0.24	2.74				Semenova et al. [34]
Ishmurzino, Russia	vicinity of abandoned mines in South Urals	7–14	Both			0.30			1.21	0.30	1.97				Semenova et al. [34]
Semenovsk, Russia	vicinity of abandoned mines in South Urals	7–14	Both			0.03			2.38	0.45	0.91				Semenova et al. [34]
Leningradskaya Oblast,’ St. Petersburg, Russia	controls from a non-urban settlement	7–9	Both	0.04		0.16	0.25	0.22	3.41	0.42	2.51			0.05	Drobyshev et al. [35]
Leningradskaya Oblast,’ St. Petersburg, Russia	proximity to the toxic waste disposal grounds	7–9	Both	0.03		0.14	0.31	0.14	2.93	0.39	3.82			0.05	Drobyshev et al. [35]
Pace del Mela, Sicily *^#^	industrial	11–14	Both	0.03		0.01	0.10		0.40	0.10	0.80			0.10	Tamburo et al. [36]
Gela, Butera and Niscemi, Sicily *^#^	polymetallic mining area	11–14	Both	0.04		0.02	0.10		0.20	0.10	0.40				Tamburo et al. [36]
Palermo, Sicily *^#^	urban	11–14	Both	0.0003		0.02	0.20		0.20	0.04	0.80			0.10	Tamburo et al. [36]
Small towns around Etna, Sicily *^#^	volcanic	11–14	Both	0.03		0.01	0.10		0.30	0.10	0.40			0.20	Tamburo et al. [36]
Sardinia, Italy	mining areas	11–13	Both	3.60		3.60	0.70		1.60	4.90	13.10			1.20	Varrica et al. [37]
Alcalá de Henares, Madrid, Spain	urban	6–9	Both	ND	ND	0.52	0.66	1.10	0.30	0.42	1.48	1.29	ND	0.44	Peña-Fernández et al. [46]
Flix, Spain	urban	12–13	Boys	0.08	0.004	ND		0.47	0.13	0.26	0.80	0.23	ND	0.19	Torrente et al. [47]
Flix, Spain	urban	12–13	Girls	0.09	0.004	0.02		0.47	0.19	0.53	0.59	0.33	ND	0.17	Torrente et al. [47]

ND = Non-detected.* Metal concentration is expressed as median. # Metal levels were measured in scalp hair.
